# Supplementary material for: Sequencing DNA methylation and hydroxymethylation at co-occurring chromatin features
Source: Nat Commun. 2026 Feb 10;17:2591. doi: 10.1038/s41467-026-69429-6 (PMC13002996; doi:10.1038/s41467-026-69429-6)
Supplement: Supplementary file 1 — Supplementary Information [file 41467_2026_69429_MOESM1_ESM.pdf]

SUPPLEMENTARY INFORMATION

Sequencing DNA methylation and hydroxymethylation at co-occurring chromatin features

Rafael de Cesaris Araujo Tavares[1], Somdutta Dhir[1], Xuan He[2], Jack Monahan[3], Minna Taipale[3], Paula Golder[3], Aldo Ciau-Uitz[3], Walraj Gosal[3], David Tannahill[1] & Shankar Balasubramanian[1,2,4,*]

[1] Cancer Research UK Cambridge Institute, University of Cambridge, Li Ka Shing Centre, Robinson Way, Cambridge, UK
[2] Yusuf Hamied Department of Chemistry, University of Cambridge, Cambridge, UK
[3] biomodal, The Trinity Building, Chesterford Research Park, Cambridge, UK
[4] School of Clinical Medicine, University of Cambridge, Cambridge, UK
** Correspondence: sb10031@cam.ac.uk (S.B.)*

Content

Supplementary Figures

Supplementary Figure 1. Detailed 6-base-seq library preparation from tagmented DNA
Supplementary Figure 2. Peak overlaps for standard CUT&Tag datasets used in this study
Supplementary Figure 3. Genomic enrichments of standard C&T for four histone modifications in E14TG2A mESCs
Supplementary Figure 4. 6-base-CUT&Tag (6B-C&T) at four major histone modifications in E14TG2A mESCs
Supplementary Figure 5. Pearson correlation analysis of enrichments obtained from 6-base-CUT&Tag and control (standard) CUT&Tag experiments
Supplementary Figure 6. Pearson correlation analysis of genome-wide modified CpG fractions from 6-base-CUT&Tag and whole-genome (untargeted) 6-base-seq
Supplementary Figure 7. Comparison between 6-base-CUT&Tag (this study) and available CUT&Tag-BS (GSE179266) data
Supplementary Figure 8. Relative abundances of 5mC and 5hmC from WG 6-base-seq and 6B-C&T at four histone modifications
Supplementary Figure 9. 5mC and 5hmC levels for different histone modifications at CpG sites shared by 6B-C&T and whole-genome 6-base-seq
Supplementary Figure 10. Differentially methylated and hydroxymethylated CpGs between 6B-C&T and whole-genome 6-base-seq
Supplementary Figure 11. Differentially methylated CpGs and differentially hydroxymethylated CpGs between 6B-C&T and whole-genome (untargeted) 6-base-seq
Supplementary Figure 12. Comparison between 6-base-CUT&Tag (this study) and ChIP-seq data used for enhancer class annotation (GSE89211)
Supplementary Figure 13. Boxplots of %5mC and %5hmC in genomic windows (IGV snapshots) shown in Main Figure 3
Supplementary Figure 14. 5mC and 5hmC levels associated with different histone marks at mESC enhancers
Supplementary Figure 15. Genomic enrichments of 6B-C&T for enhancer-associated histone modifications at annotated enhancer types
Supplementary Figure 16. Distributions of 5mC and 5hmC levels at different histone marks in mESC enhancers

Supplementary Figure 17. Architecture of machine learning model used for enhancer classification
Supplementary Figure 18. Cross-dataset validation of 6B-C&T vs. WG 6B-seq machine learning models for different enhancer types

Supplementary references

Supplementary Figures

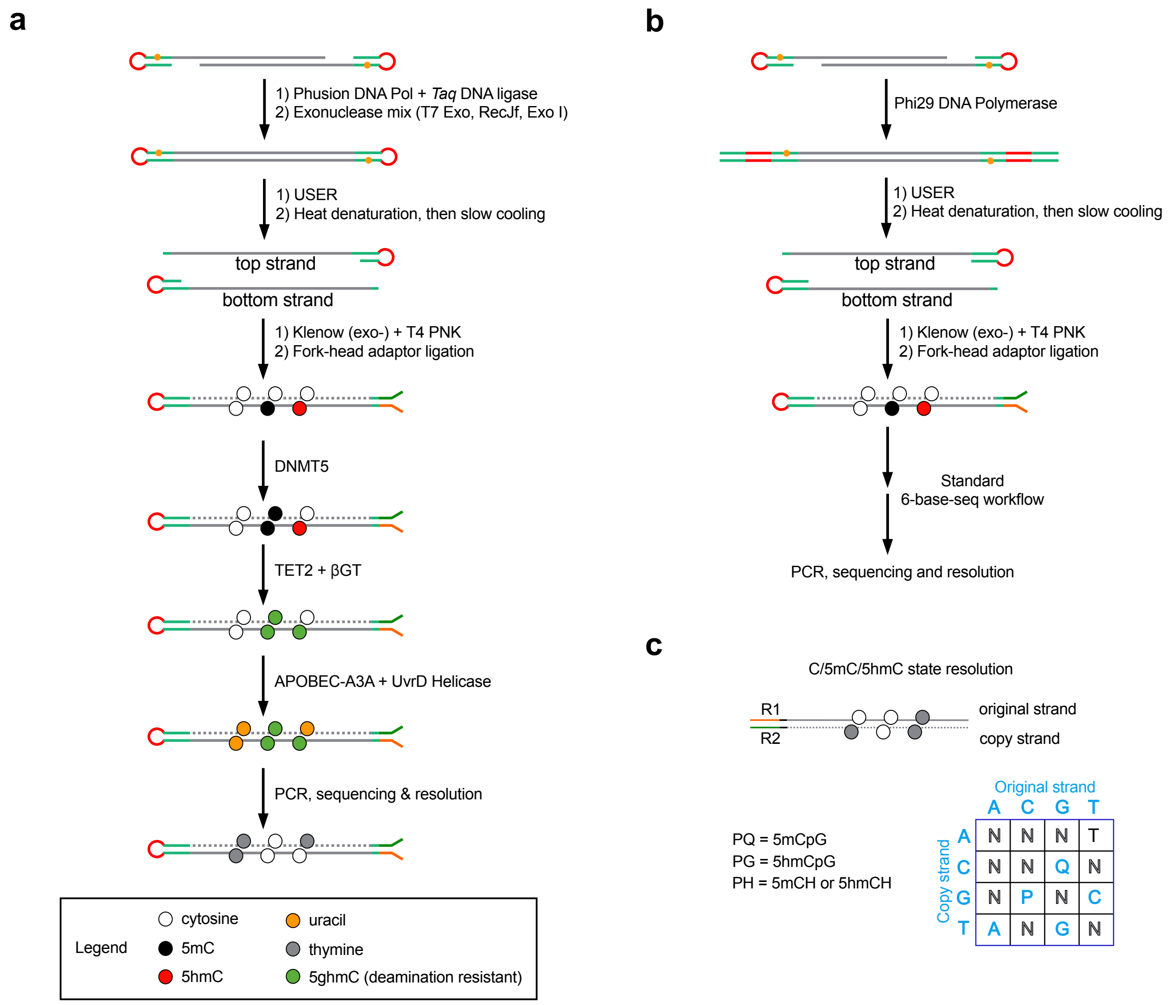

Supplementary Figure 1. Detailed 6-base-seq library preparation from tagmented DNA. (a) Workflow including DNA circularization and exonuclease treatment. For clarity, subsequent enzymatic steps are shown only for the bottom strand of the initial dsDNA fragment. DNA is first circularised by gap filling with Phusion DNA polymerase and nicks sealed with Taq DNA ligase. Subsequent exonuclease treatment specifically digests linear DNA while dumbbell-like fragments are protected. Uracil-specific excision reagent (USER) cleavage then generates a single nucleotide gap at both uracil sites present in each hairpin. This allows for strand separation of the original duplex via heat denaturation and hairpin re-folding on each strand via slow cooling. Klenow (3' → 5' exo–) next synthesises a copy strand on each individual strand. T4 polynucleotide kinase (PNK) is included for gap repair of USER-digested sites. A "fork-head" adaptor is then ligated to the free end of double-strand DNA fragments. DNMT5, chosen for its high specificity of copy methylation over de novo methylation, then specifically methylates the CpG on the recently synthesised copy strand only when the original strand contains a 5mCpG[1]. TET2 is next used to oxidise all 5mC groups to 5hmC followed by their protection in the same reaction by glycosylation using beta-glucosyltransferase (βGT). Finally, APOBEC3A (ssDNA deaminase) converts all unprotected cytosines to uracils, assisted by the dsDNA unwinding activity of the UvrD helicase. Primers complementary to the fork-head adaptor sequences and containing Illumina P5 and P7 sequences are then used to amplify all fragments by PCR to generate a library for Illumina sequencing. All cytosine states are schematically represented and colour-coded according to the legend. "5mC" = 5-methylcytosine; "5hmC" = 5-hydroxymethylcytosine; "5ghmC" = 5-glucosylhydroxymethylcytosine. **(b)** Workflow without

DNA circularization/exonuclease treatment. Phi29 DNA Pol is used for gap filling and to copy the original hairpin sequence, generating a dsDNA intermediate that is subsequently USER-digested and subjected to the same downstream enzymatic steps as in **a**. **(c)** 6-base-seq base resolution rules.

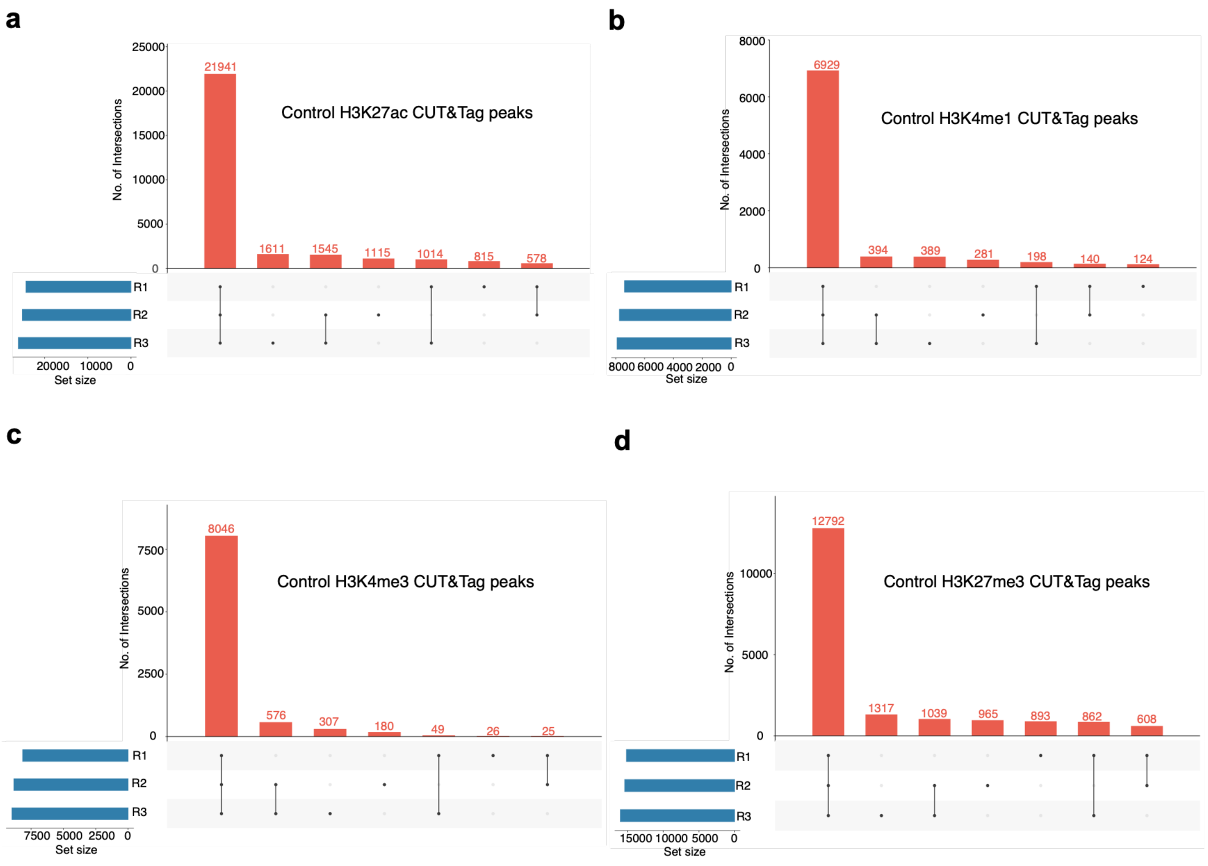

Supplementary Figure 2. Peak overlaps for standard CUT&Tag data used in this study. UpSet plots representing the number of genomic regions (red bars) identified as enriched (i.e., peaks) in each CUT&Tag experiment for **(a)** H3K27ac, **(b)** H3K4me1, **(c)** H3K4me3 and **(d)** H3K27me3. The number of peak regions shared across multiple experimental replicates is indicated by a line joining the dots corresponding to each replicate. The total number of peaks (set size) for each replicate is shown on the bottom left (horizontal blue bars).

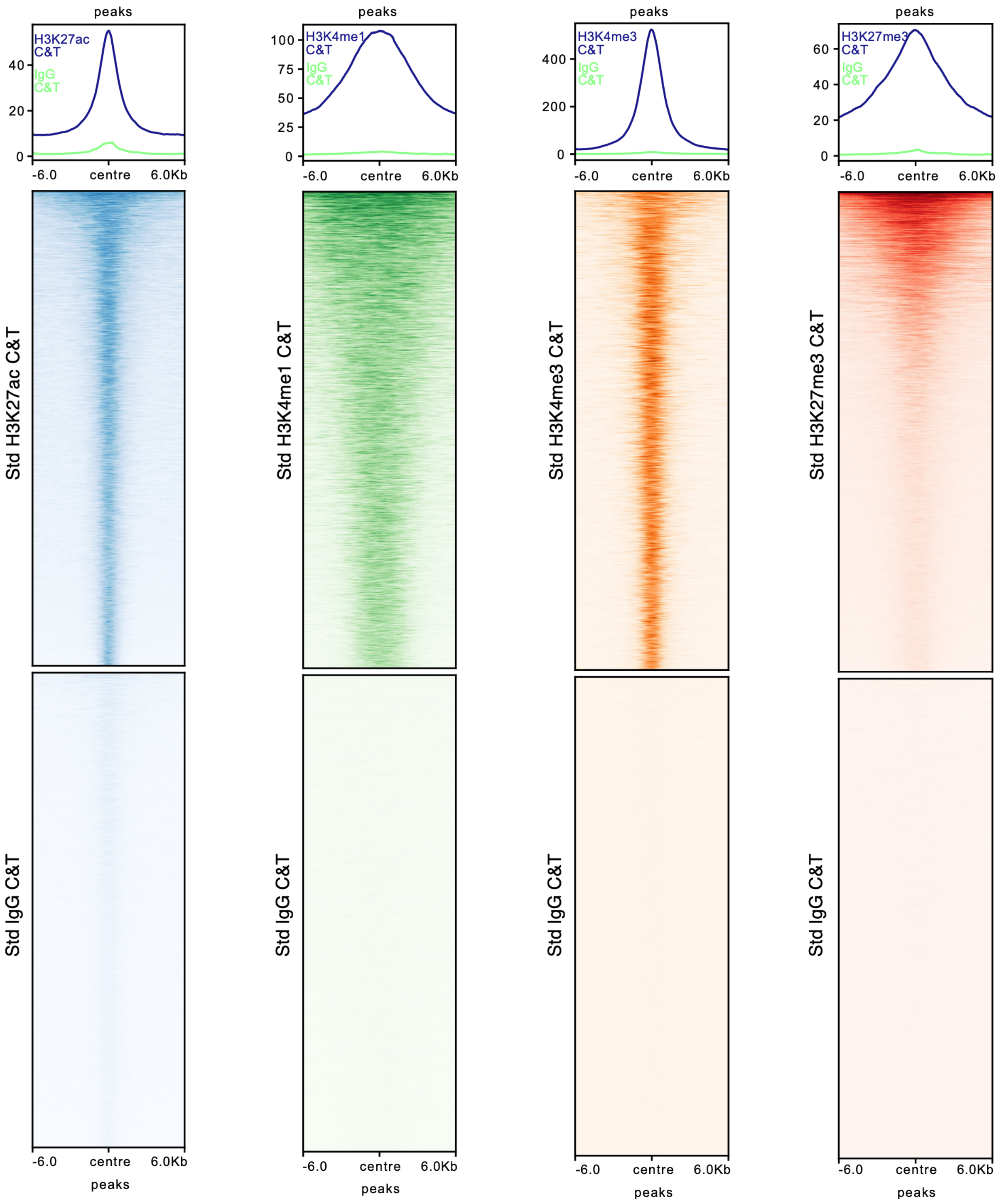

Supplementary Figure 3. Genomic enrichments of standard C&T for four histone modifications in E14TG2A mESCs. Genomic heatmaps for each histone mark (top) are plotted in +/- 6 kilobase windows relative to the centre of each peak region, along with an IgG control (bottom). H3K27ac data is shown in blue, H3K4me1 in green, H3K4me3 in orange and H3K27me3 in red. Metagene enrichment plots for the same windows are displayed above each heatmap (histone mark C&T line in blue and IgG control in green). Results from a representative 6B-C&T experiment for each mark are shown (H3K27me3, N = 2; H3K27ac, N = 3; H3K4me1, N = 3; H3K4me3, N = 3; IgG, N = 3; N = biologically independent replicates).

a

b

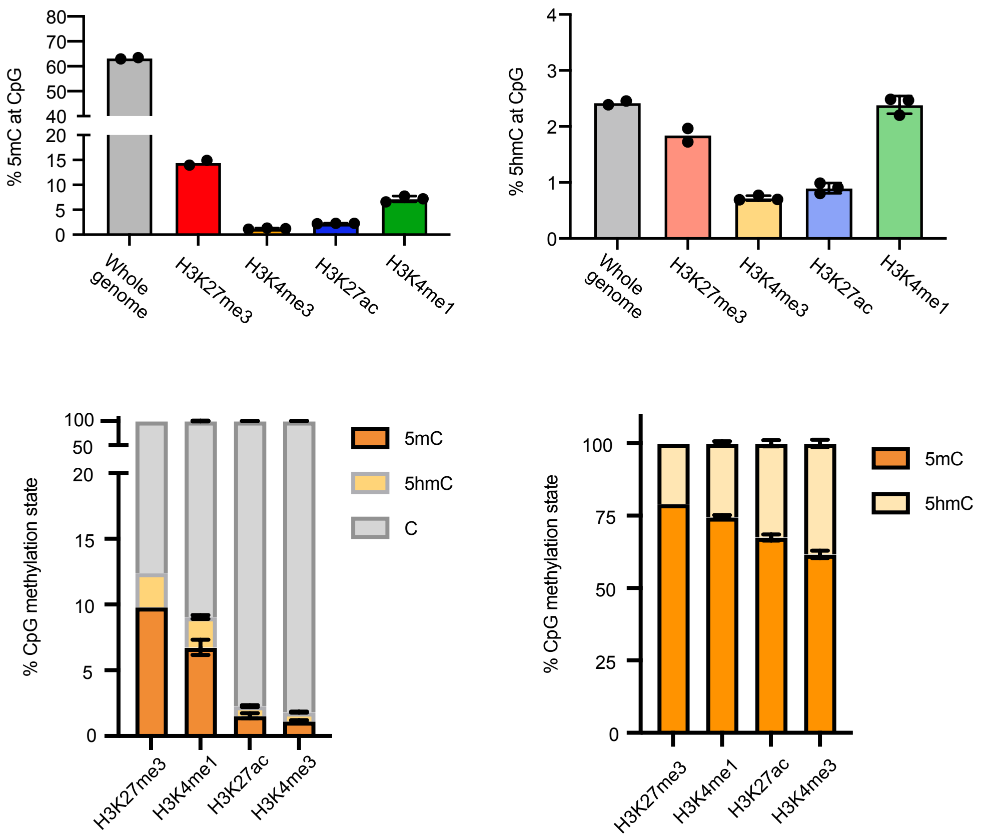

Supplementary Figure 4. 6-base-CUT&Tag (6B-C&T) at four major histone modifications in E14TG2A mESCs. (a) Average (mean) genome-wide percentages of 5mC (left) and 5hmC (right) for four histone marks (red: H3K27me3, N = 2; orange: H3K4me3, N = 3; blue: H3K27ac, N = 3; green: H3K4me1, N = 3) by 6B-C&T in E14TG2A mESCs. Whole-genome levels by untargeted 6-base sequencing are shown in grey (N = 2). Error bars indicate standard deviation when appropriate and data is shown for all biologically independent experiments. **(b)** Average (mean) distribution of CpG methylation states at each histone mark. Left: stacked bar plot for all three CpG states (C, 5mC and 5hmC) at each histone mark. Right: quantification of the relative amounts of 5mC and 5hmC in each case. Replicate information as in **a**.

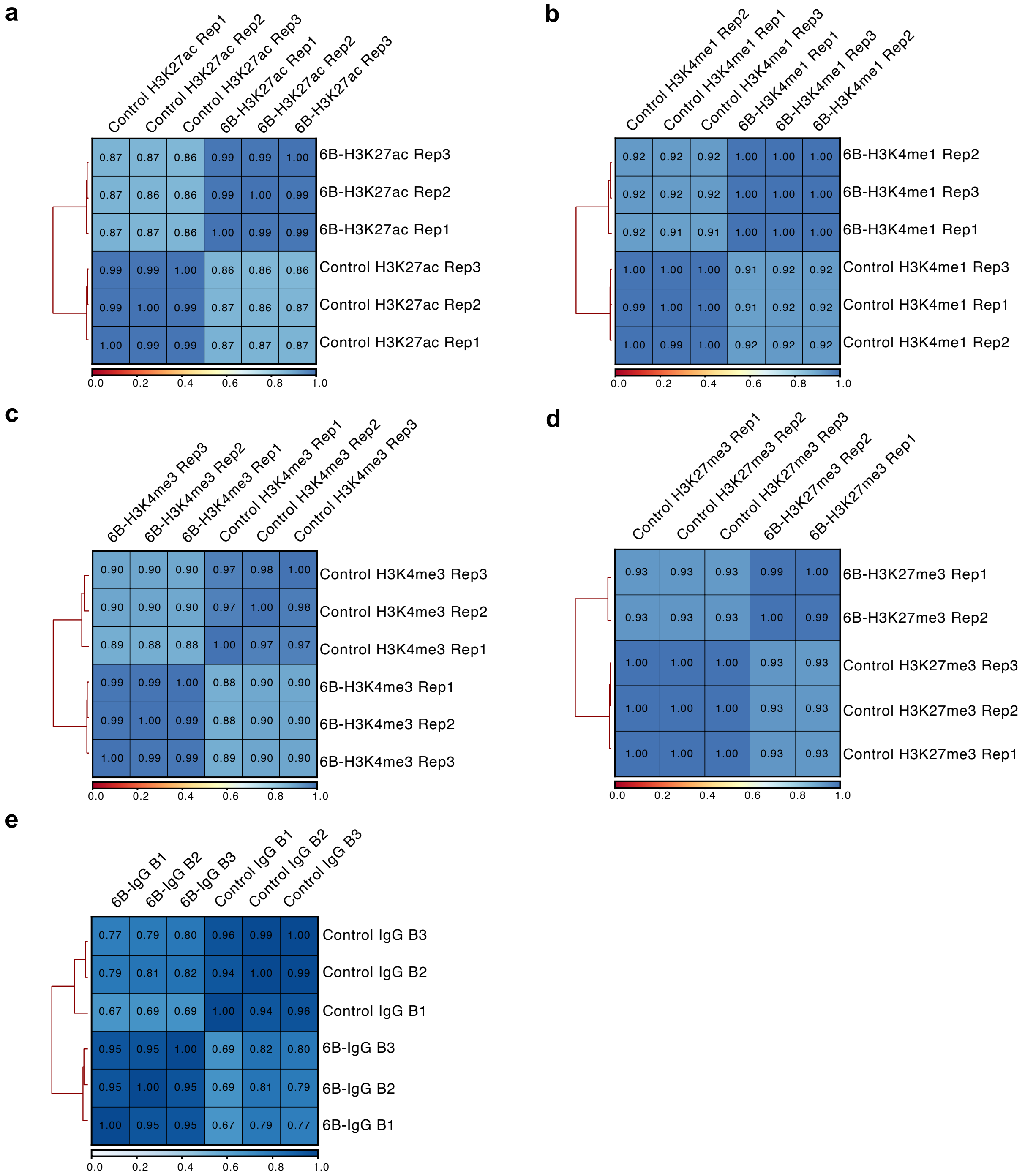

Supplementary Figure 5. Pearson correlation analysis of enrichments for 6-base-CUT&Tag and control (standard) CUT&Tag experiments. Correlation matrices for all experimental replicates of both experiments are shown for the four histone marks used in this study: **(a)** H3K27ac 6B-CUT&Tag vs. control H3K27ac CUT&Tag, **(b)** H3K4me1 6B-CUT&Tag vs. control H3K4me1 CUT&Tag, **(c)** H3K4me3 6B-CUT&Tag vs. control H3K4me3 CUT&Tag, **(d)** H3K27me3 6B-CUT&Tag vs. control H3K27me3 CUT&Tag and **(e)** IgG 6B-CUT&Tag vs. control IgG CUT&Tag. Pearson correlation coefficient values for each pairwise comparison are displayed and colour-coded according to the legend under each matrix.

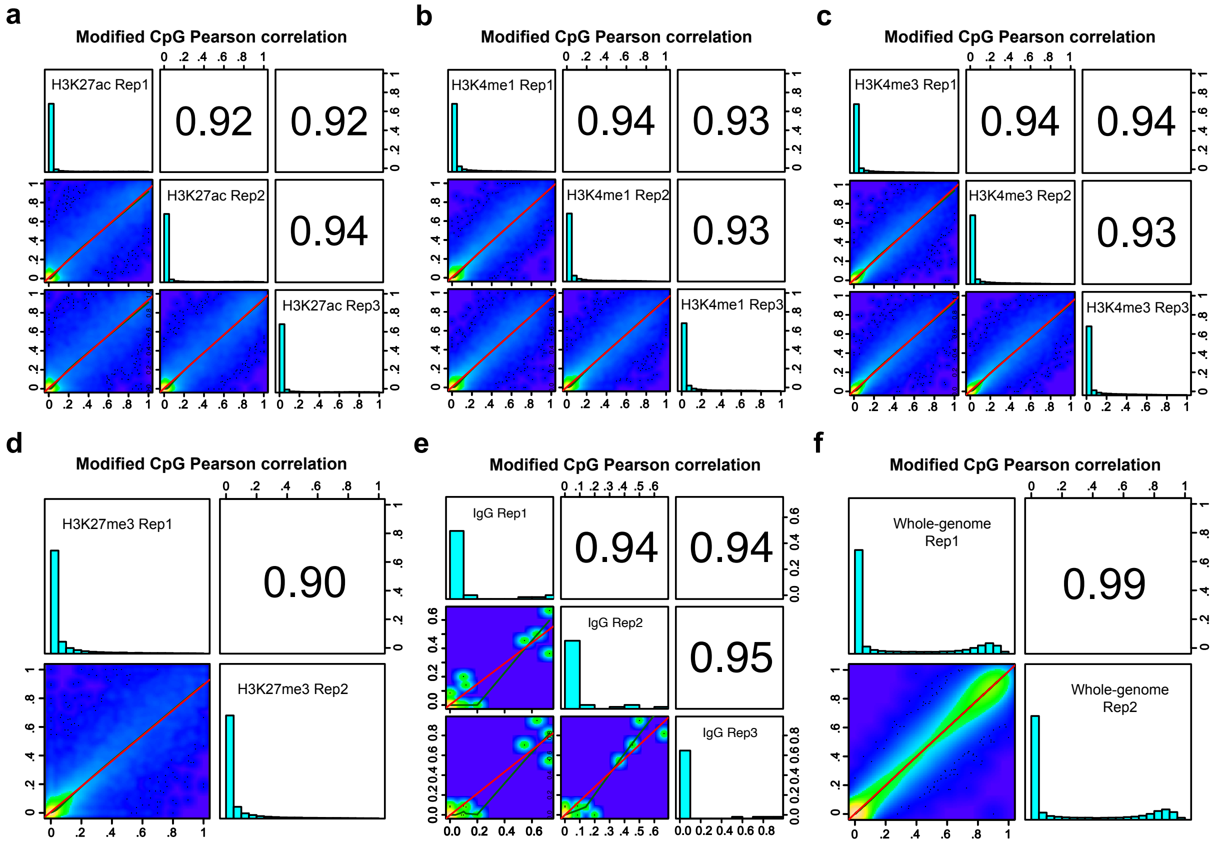

Supplementary Figure 6. Pearson correlation analysis of genome-wide modified CpG fractions for 6-base-CUT&Tag and whole-genome (untargeted) 6-base-seq. Histograms of modified CpG fractions over 200-bp genomic tiles from each replicate of different 6-base CUT&Tag **(a-e)** and whole-genome 6-base-seq **(f)** experiments. Correlation scatter plots and Pearson correlation coefficients are shown for each pairwise comparison.

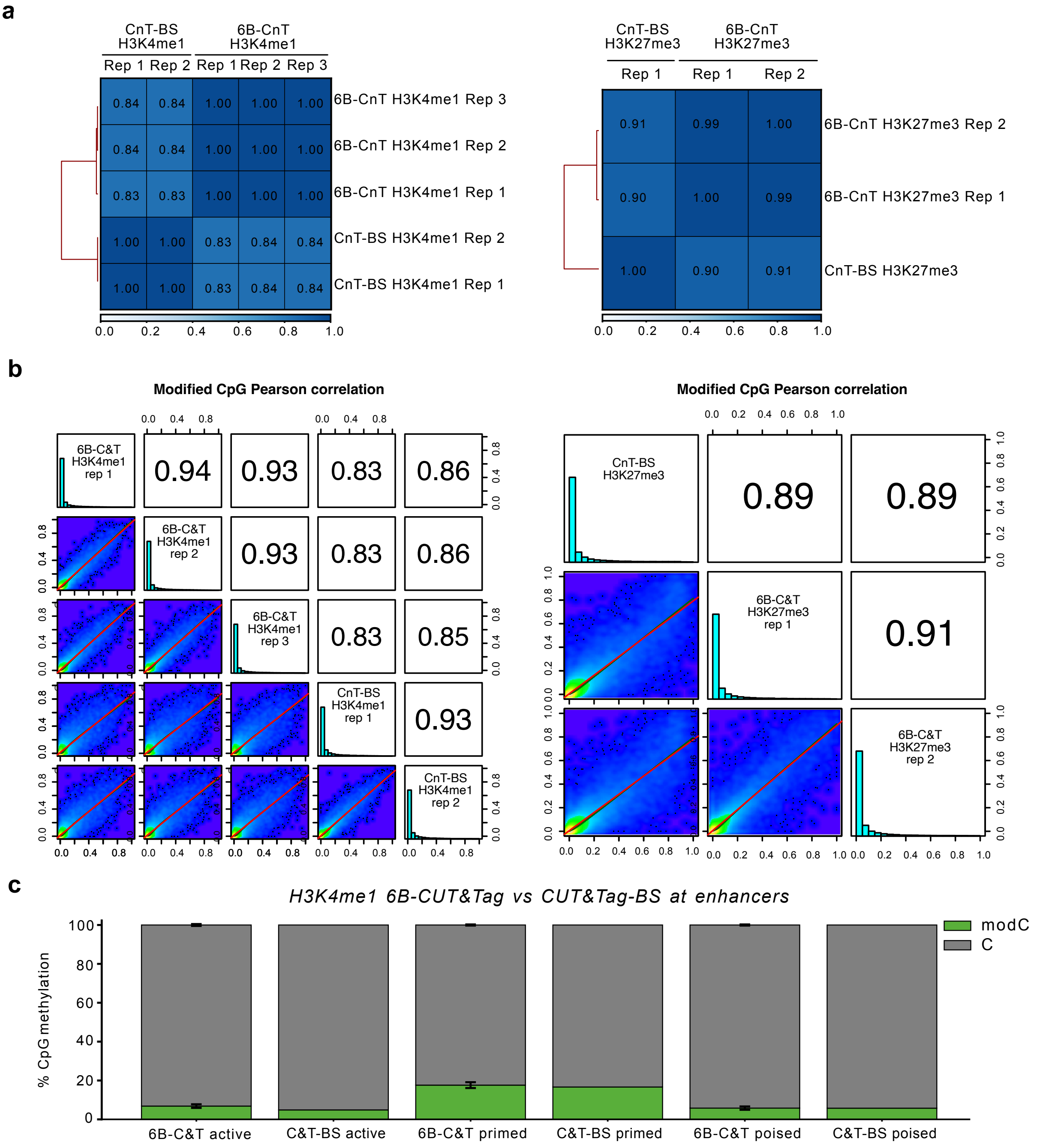

Supplementary Figure 7. Comparison between 6-base-CUT&Tag (this study) and available CUT&Tag-BS[2] (GSE179266) data. (a) Pearson correlation matrices of genomic fragment enrichments for 6B-C&T compared to a publicly available C&T-BS dataset for two histone marks: H3K4me1 (left) and H3K27me3 (right). **(b)** Correlation matrices for genome-wide total DNA methylation (modC) fractions for the same datasets (left, H3K4me1; right, H3K27me3). Histograms of modified CpG fractions for 200-bp genomic tiles from each available experimental replicate are shown on the diagonal alongside scatter plots below the diagonal. Pearson correlation coefficients are displayed for each pairwise comparison above the diagonal. **(c)** Comparison of total DNA methylation (modC) from H3K4me1 6B-C&T and H3K4me1 C&T-BS at enhancers. Stacked bar plots show mean % modC (green) and % C (grey) for different sets of annotated enhancer regions. Error bars represent standard deviation when appropriate (3 independent experiments shown for 6B-C&T; 2 independent experiments shown for C&T-BS).

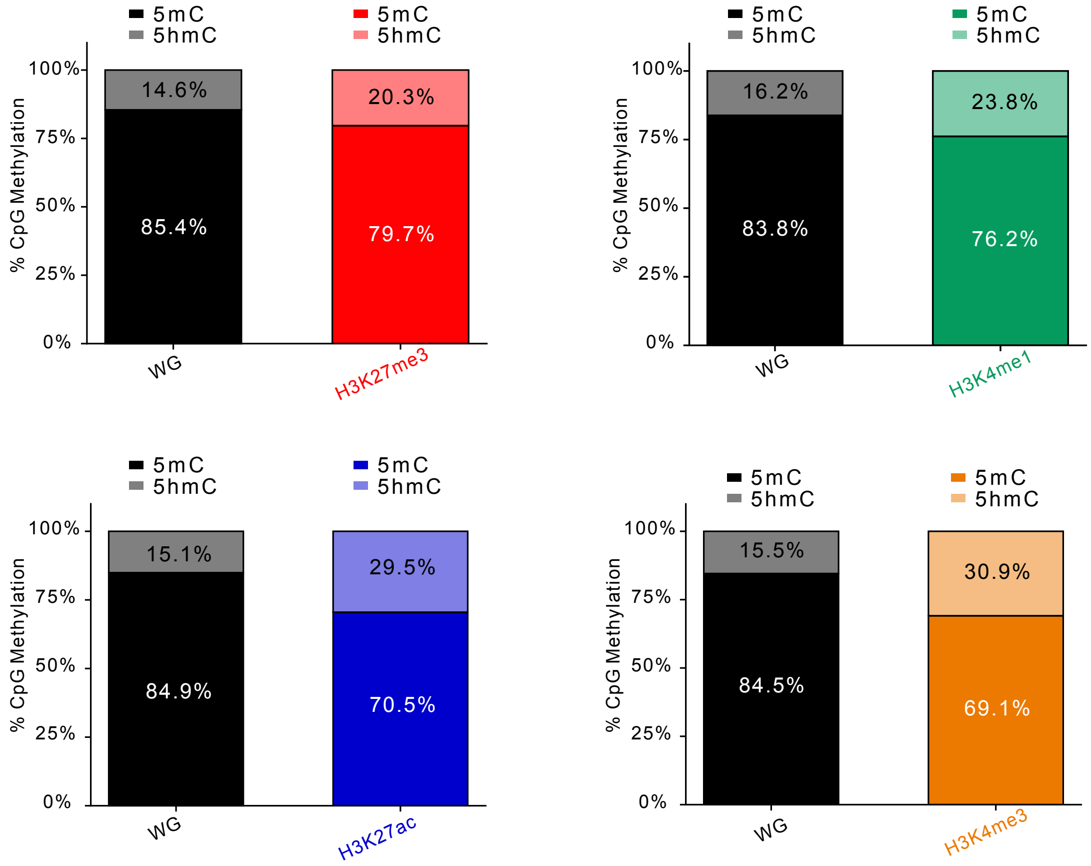

Supplementary Figure 8. Relative abundances of 5mC and 5hmC from WG 6-base-seq and 6B-C&T at four histone modifications. In each graph, stacked bar plots show WG and 6B-C&T at CpG sites commonly detected by both experiments. 5mC is represented in dark and 5hmC in light colours. Percentages of each base are annotated for each condition. Results from a representative 6B-C&T experiment for each mark and WG 6B-seq are shown (H3K27me3, N = 2; H3K4me1, N = 3; H3K27ac, N = 3; H3K4me3, N = 3; WG, N = 2; N = biologically independent replicates).

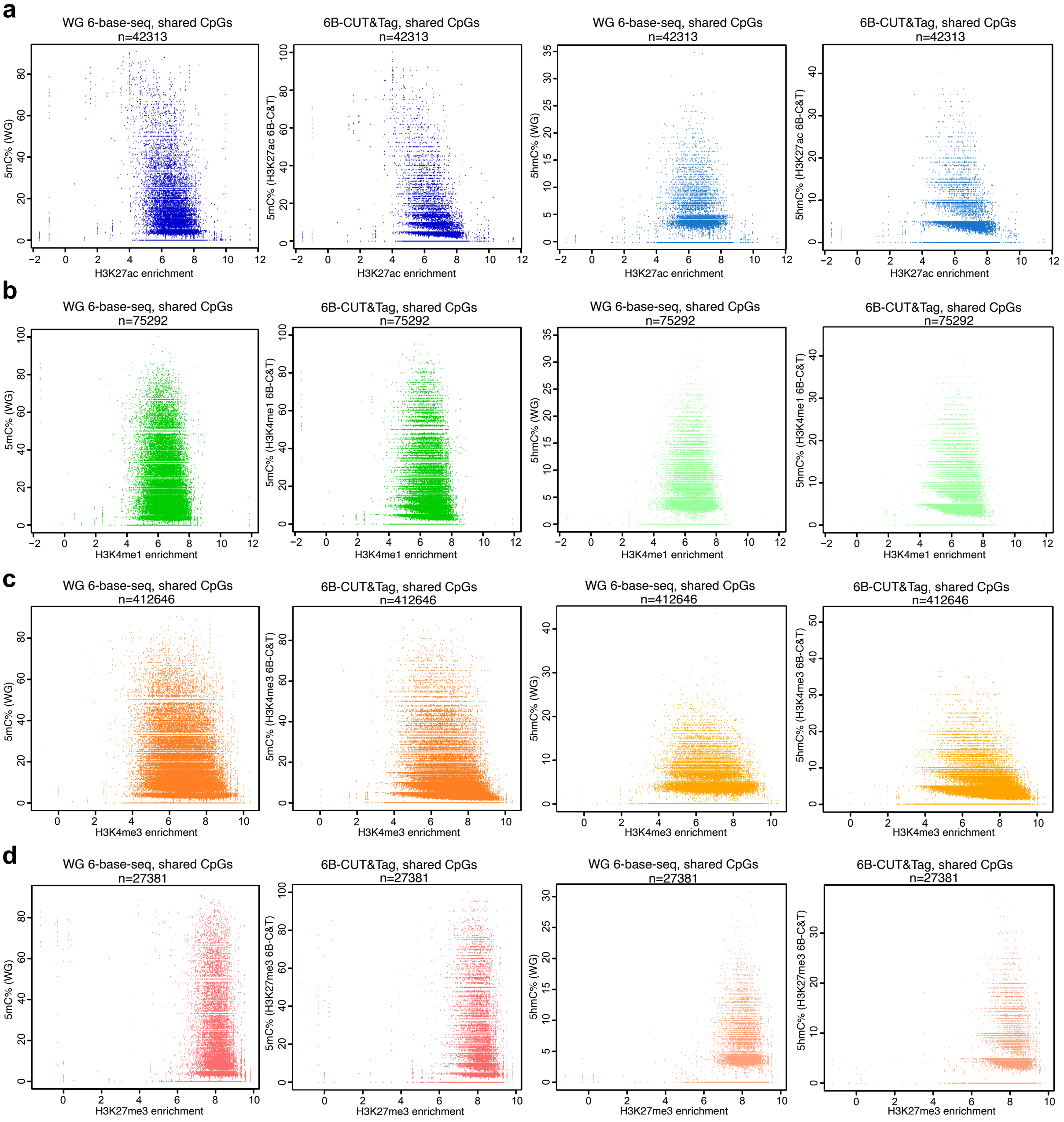

Supplementary Figure 9. 5mC and 5hmC levels for different histone modifications at CpG sites shared by 6B-C&T and whole-genome 6-base-seq. Each graph shows percentage 5mC or 5hmC for CpG sites present in both whole genome 6-base-seq and 6B-C&T data (shared). Cytosine modification percentages from each dataset are plotted as a function of histone mark enrichment (log_2 scale) for each of the four profiled marks: **(a)** H3K27ac, **(b)** H3K4me1, **(c)** H3K4me3 and **(d)** H3K27me3. “n” in each plot is the number of shared CpG sites identified in each analysis. Results are representative of multiple biologically independent replicates (H3K27me3, N = 2; H3K27ac, N = 3; H3K4me1, N = 3; H3K4me3, N = 3; WG, N = 2).

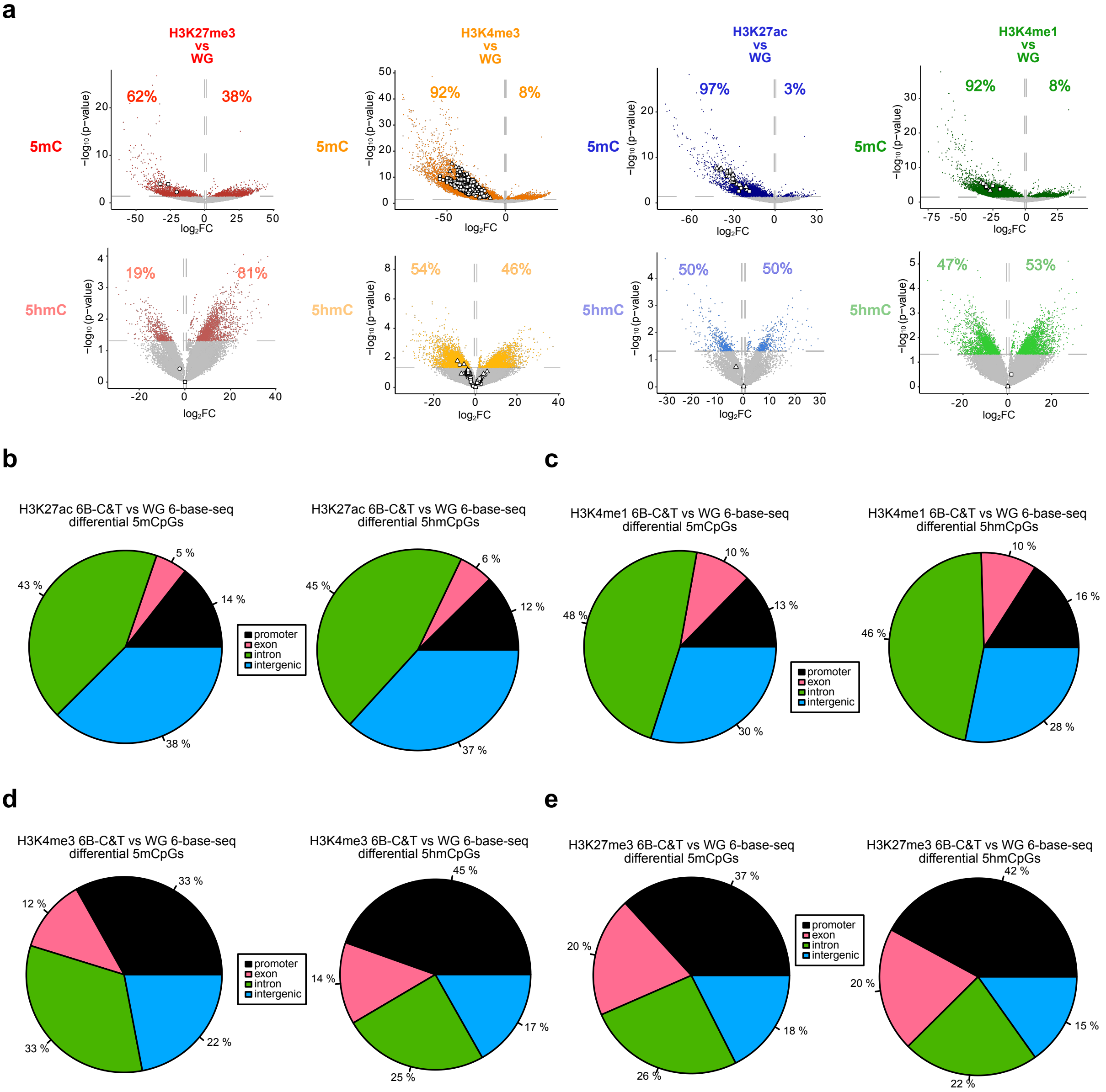

Supplementary Figure 10. Differentially methylated and hydroxymethylated CpGs between 6B-C&T and whole-genome 6-base-seq. (a) Volcano plots of differentially methylated 5mCpGs (DMCs, top) and 5hmCpGs (DHMCs, bottom) for each 6B-C&T dataset relative to whole-genome 6-base-seq (WG). Differentially methylated sites (p-value < 0.05, $|\log_2 FC| \geq 0.5$) are coloured for each histone mark. Each plot compares histone mark-specific values (6B-C&T) relative to WG 6-base-seq data. Differential methylation was computed from biologically independent experiments (red: H3K27me3, N = 2; orange: H3K4me3, N = 3; blue: H3K27ac, N = 3; green: H3K4me1, N = 3; WG, N = 2). Non-significant changes are shown in grey. The percentage of significant differences where 6B-C&T values are lower (left) or higher (right) than in whole-genome data are indicated in each plot. CpG sites at example promoter regions showing allele-specific expression in E14TG2A mESCs (imprinted loci) are annotated in each plot (*Igf2r* = white filled circles; *Kcnq1ot* = white filled squares; *Peg3* = white filled triangles). **(b)** Pie chart of genomic element distributions of differential 5mCpGs and 5hmCpGs (6B-C&T vs WG) for H3K27ac. Analogous pie charts are shown in **(c)** for H3K4me1, **(d)** for H3K4me3 and **(e)** for H3K27me3.

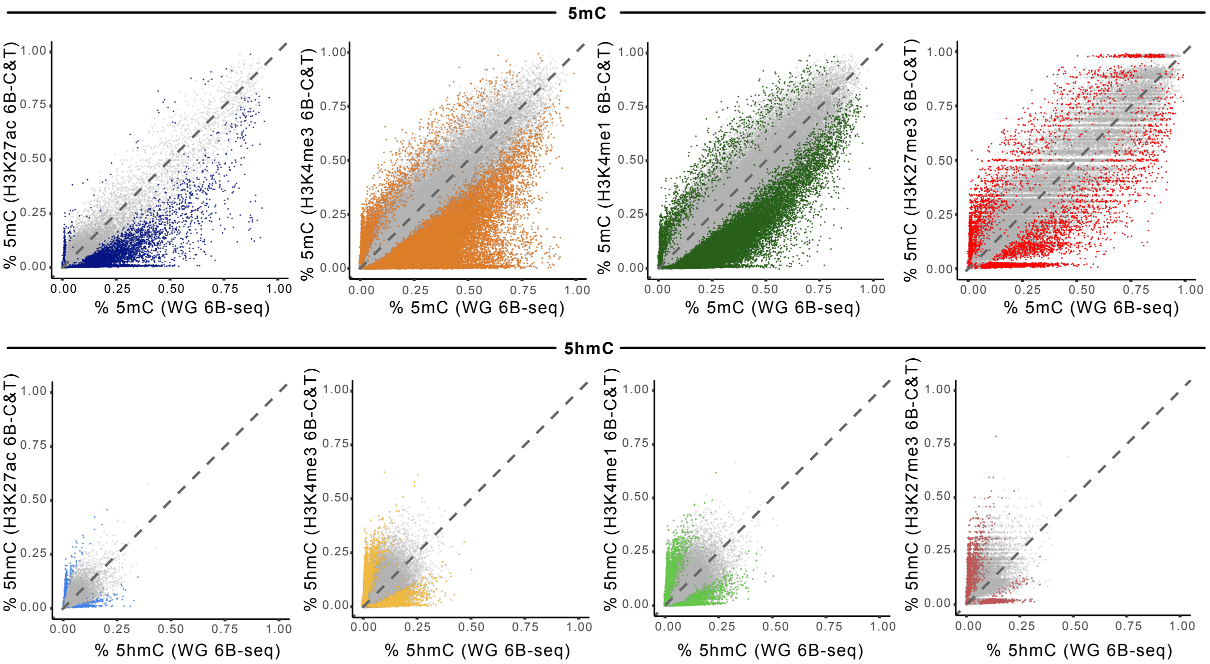

Supplementary Figure 11. Differentially methylated CpGs and differentially hydroxymethylated CpGs between 6B-C&T and whole-genome (untargeted) 6-base-seq. Scatter plots of %5mC (top row) and %5hmC (bottom row) for CpG sites (round dots) captured in both 6B-C&T and whole-genome (WG) 6-base-seq. In each plot, the y-axis coordinate of each CpG site is obtained from 6B-C&T data (replicate-averaged) and the x-axis coordinate is obtained from WG data (replicate-averaged). Differentially methylated sites (p-value < 0.05, $|\log_2 FC| \geq 0.5$) between both methods are coloured for each histone mark (H3K27ac in blue, H3K4me3 in orange, H3K4me1 in green and H3K27me3 in red). Non-significant changes are shown in grey. Differential methylation was computed from biologically independent replicates (H3K27me3, N = 2; H3K4me1, N = 3; H3K27ac, N = 3; H3K4me3, N = 3; WG, N = 2; N = biologically independent replicates).

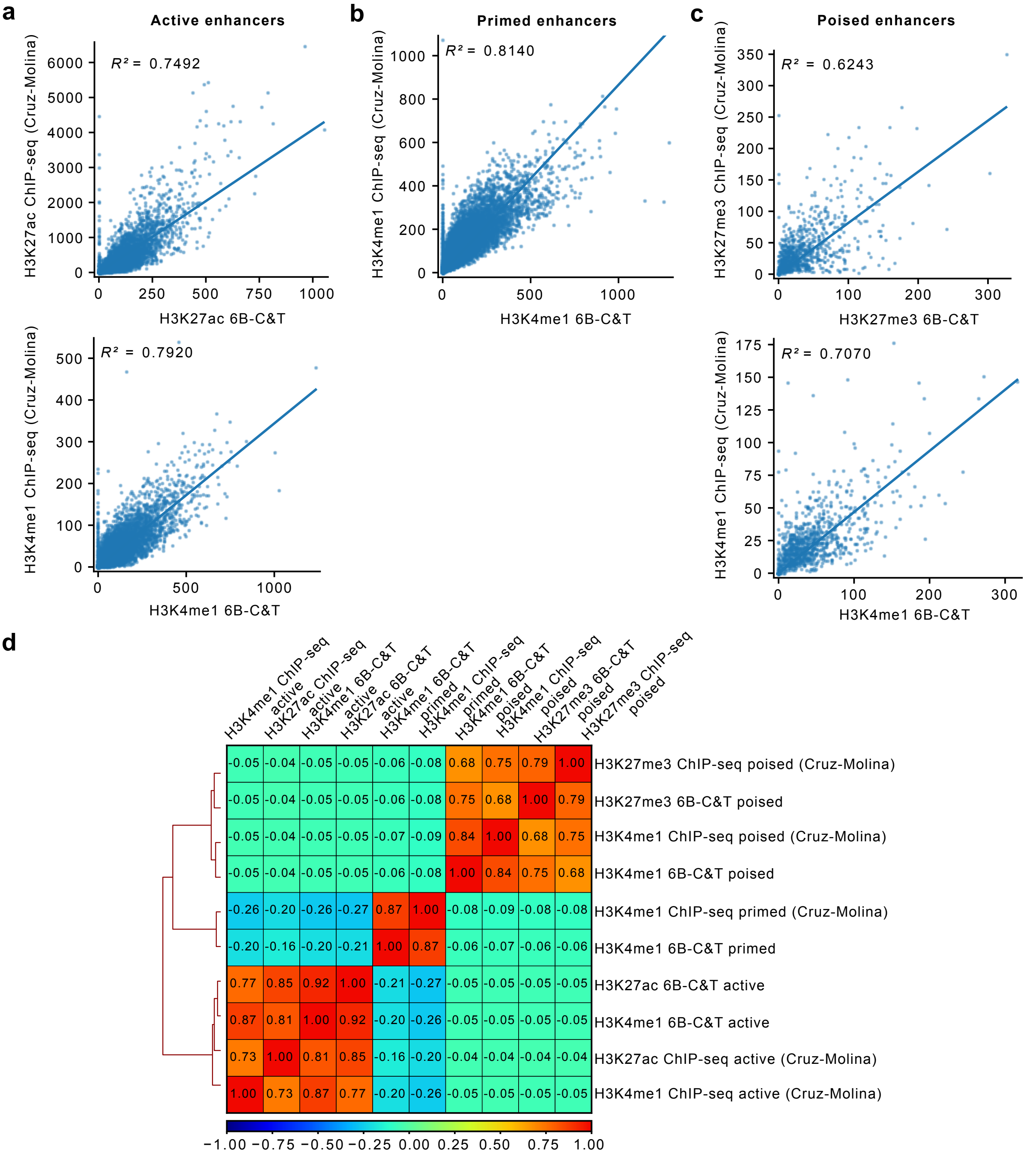

Supplementary Figure 12. Comparison between 6-base-CUT&Tag (this study) and ChIP-seq data used for enhancer class annotation (GSE89211)[3]. Correlation scatter plots for ChIP-seq data from Cruz-Molina et al. (2017) and 6B-C&T data for enhancer-associated histone marks are plotted for different sets of annotated enhancer regions: **(a)** active enhancers (H3K27ac and H3K4me1), **(b)** primed enhancers (H3K4me1) and **(c)** poised enhancers (H3K27me3 and H3K4me1). In all cases, CPM-normalised genomic enrichments (bin size = 10 bp) are plotted for each experiment (y-axis = ChIP-seq and x-axis = 6B-C&T) and R-squared (R^2) values are annotated on each plot. Least squares polynomial (first-degree) fitting was performed with *numpy.polyfit* to produce the fitting line. A single replicate from each experiment was used as representative of the overall trend (6B-C&T: H3K27me3, N = 2; H3K4me1, N = 3; H3K27ac, N = 3; ChIP-seq: N = 1 for each histone mark, as available in GSE89211). **(d)** Pearson correlation matrix for data plotted in **a-c**. Pearson correlation coefficients (Pearson's R) are shown for each pairwise comparison.

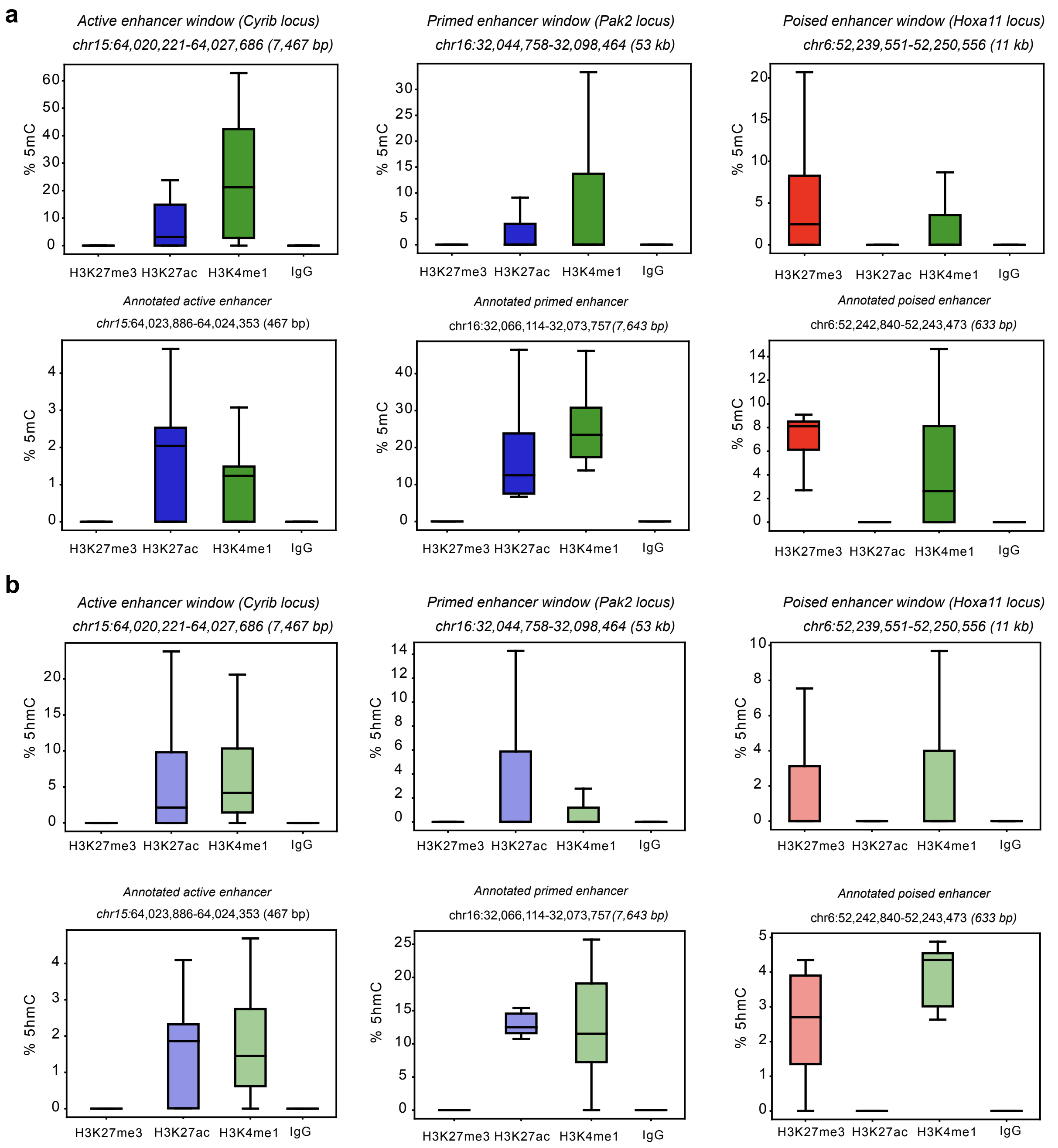

Supplementary Figure 13. Boxplots of %5mC and %5hmC in genomic windows (IGV snapshots) shown in Main Figure 3. (a) Boxplots of %5mC from 6B-C&T at enhancer marks (H3K27me3 in red, H3K27ac in blue and H3K4me1 in green) and IgG control (black) for active (left column), primed (centre column) and poised (right column) enhancer loci. The top panels show the full genomic windows in Main Figure 3 and the bottom panels show only the annotated enhancer window in each case. **(b)** Same as in **a** but for %5hmC. Whiskers represent Q3 + 1.5 x interquartile range (upper) and Q1 – 1.5 x interquartile range (lower).

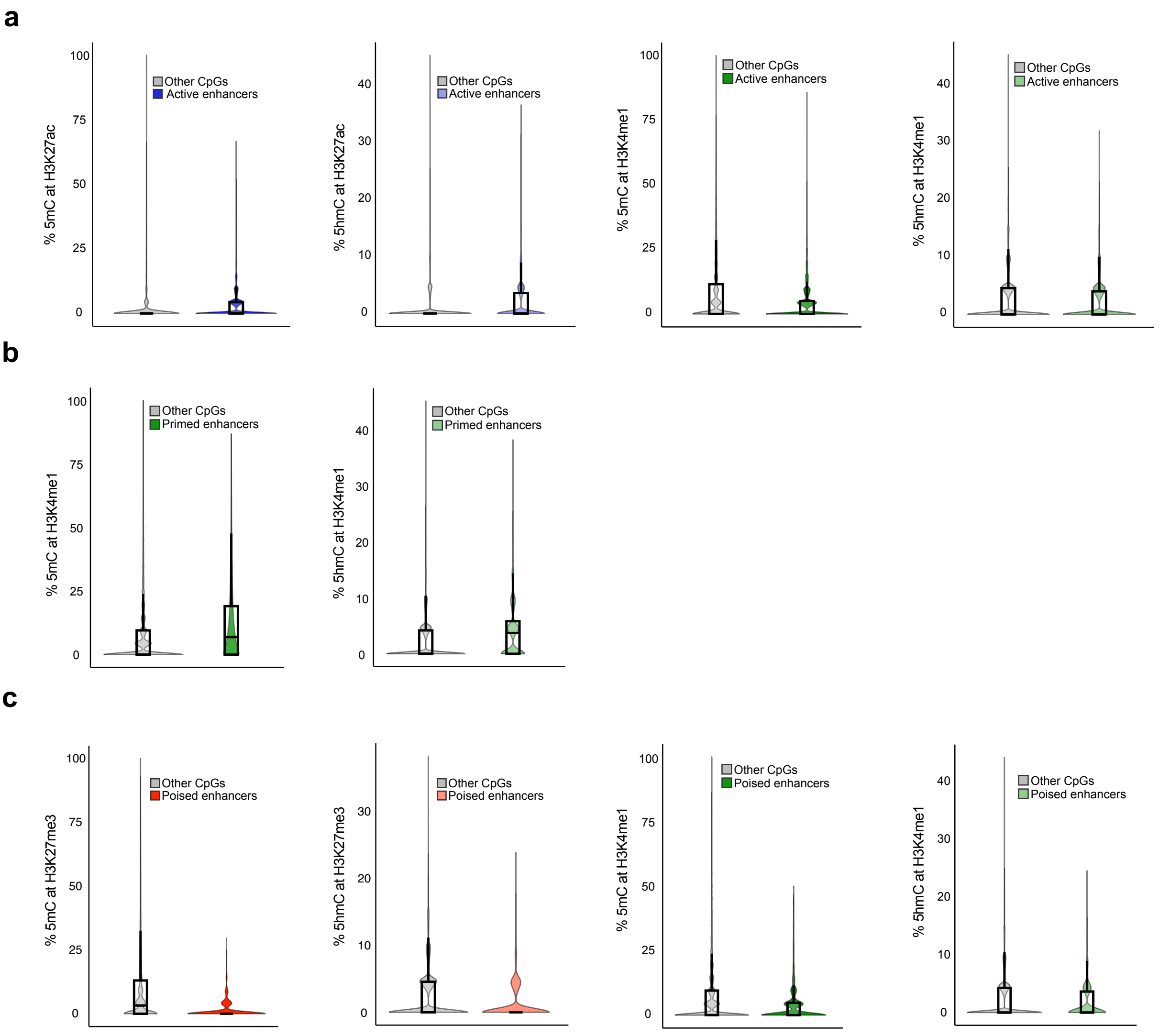

Supplementary Figure 14. 5mC and 5hmC levels associated with different histone marks at mESC enhancers. (a) Percentage 5mC and 5hmC at CpGs at active enhancers vs CpGs outside active enhancers (labelled "other CpGs"). H3K27ac 6B-C&T data (blue vs grey) and H3K4me1 6B-C&T data (green vs grey) are shown for this enhancer type. **(b)** Percentage 5mC and 5hmC at CpGs at primed enhancers vs other CpGs. H3K4me1 6B-C&T data (green vs grey) is plotted for both groups of CpGs in each case. **(c)** Percentage 5mC and 5hmC at CpGs at poised enhancers vs other CpGs. H3K27me3 6B-C&T data (red vs grey) and H3K4me1 6B-C&T data (green vs grey) are shown for this enhancer type. All distributions are represented with violin plots (light grey) accompanied by boxplots (black) to indicate the median, interquartile range and upper bound values (3rd quartile + 1.5 x interquartile range). Descriptive statistics and Wilcoxon rank sum test results are listed in Supplementary Data 1 – Table 1. Results from a representative 6B-C&T experiment for each mark are shown (red: H3K27me3, N = 2; blue: H3K27ac, N = 3; green: H3K4me1, N = 3; N = biologically independent replicates).

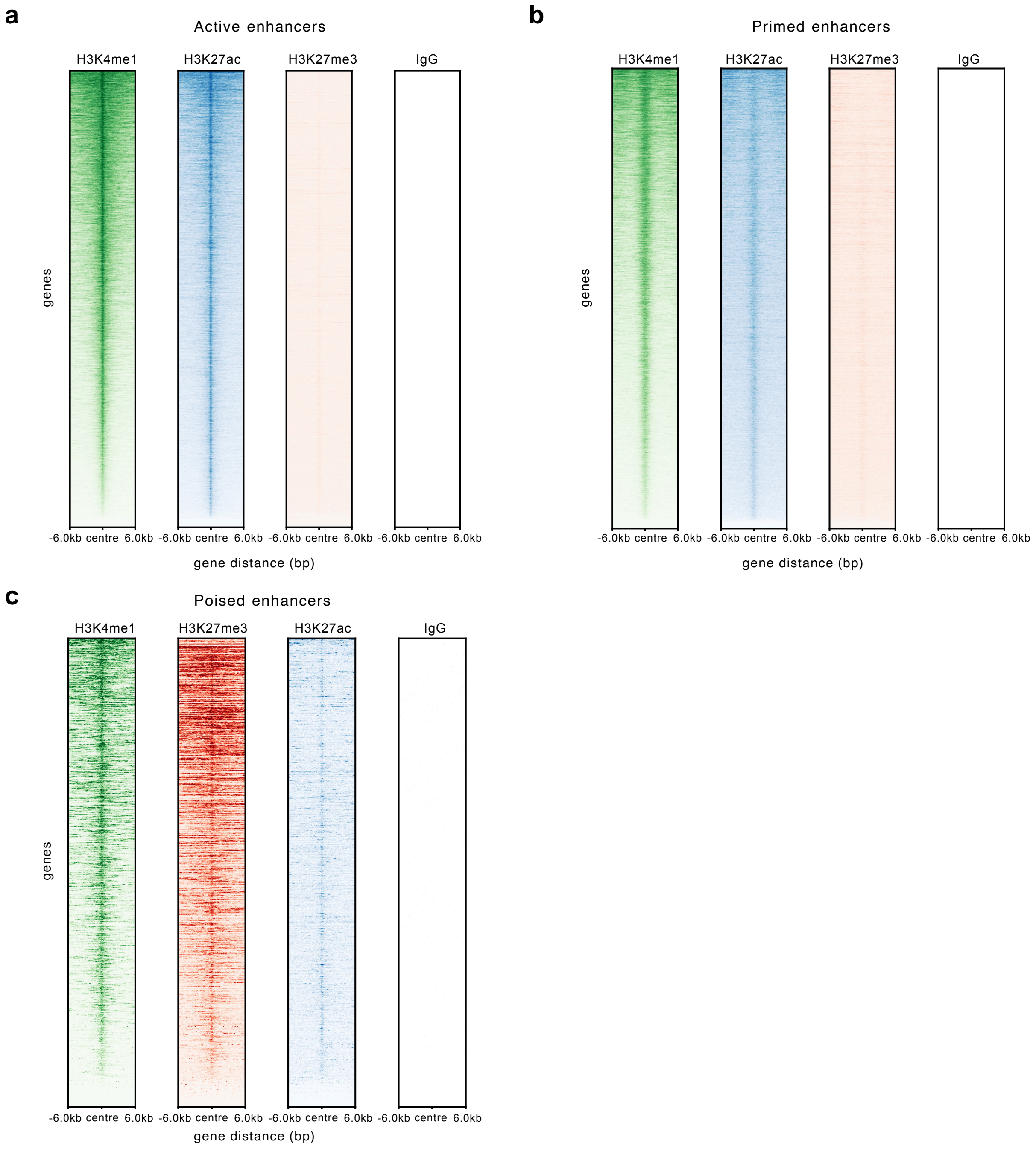

Supplementary Figure 15. Genomic enrichments of 6B-C&T for enhancer-associated histone modifications at annotated enhancer types. Genomic heatmaps for each histone mark (H3K4me1 in green; H3K27ac in blue; H3K27me3 in red and IgG control in grey) are plotted in +/- 6 kilobase windows relative to the centre of each enhancer locus for a set of annotated **(a)** active, **(b)** primed and **(c)** poised enhancers. Results from a representative 6B-C&T experiment for each mark are shown (H3K27me3, N = 2; H3K27ac, N = 3; H3K4me1, N = 3; IgG, N = 3; N = biologically independent replicates).

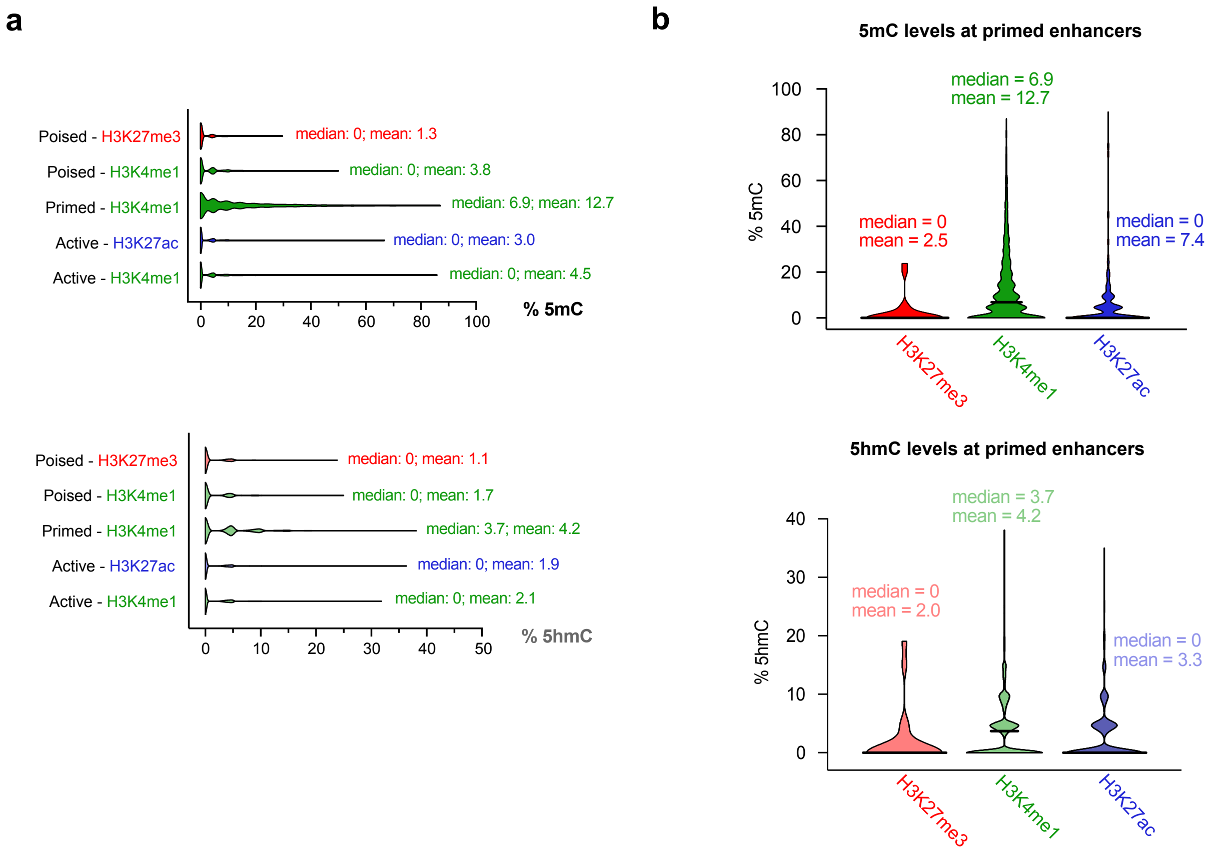

Supplementary Figure 16. Distributions of 5mC and 5hmC levels at different histone marks in mESC enhancers. (a) Global distributions of 5mC (top) and 5hmC (bottom) percentages in annotated mESC enhancer regions for the major histone modification(s) associated with each enhancer type. Average percentage (median and mean) of 5mC and 5hmC at each histone mark are shown. **(b)** Violin plots of 5mC (top) and 5hmC (bottom) levels at primed enhancers for H3K27me3 (red), H3K4me1 (green) and H3K27ac (blue). The median (50th percentile) of each distribution is shown as a horizontal bar (black) and both median and mean values are annotated. Data from one representative biological replicate is shown in all plots (H3K27me3, N = 2; H3K27ac, N = 3; H3K4me1, N = 3; N = biologically independent replicates).

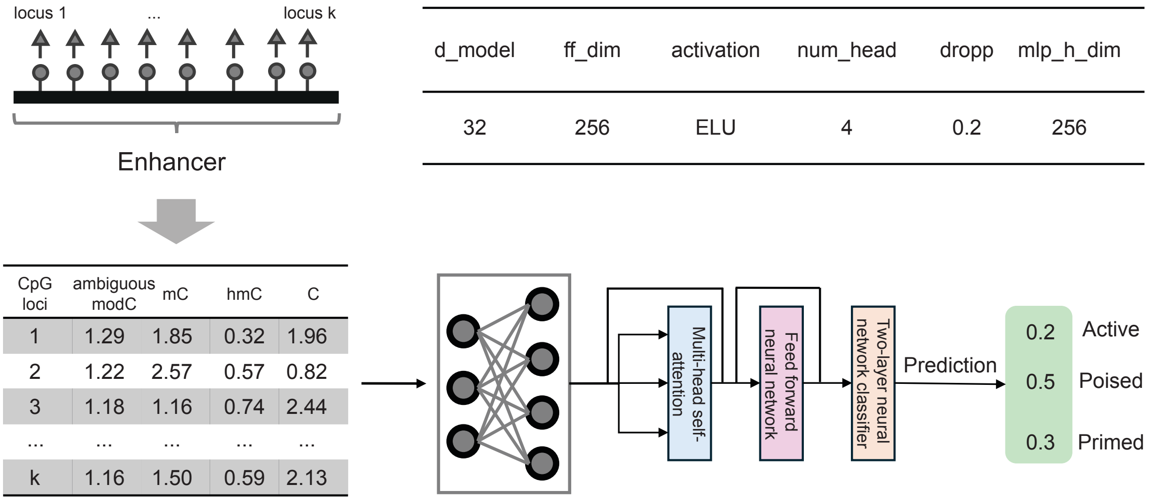

Supplementary Figure 17. Architecture of machine learning model used for enhancer classification. For each enhancer, normalised counts representing the different methylation states (ambiguous_modC, mC, hmC, C) across sequential CpG sites were organized into an input vector. This input was first processed by a linear projection layer, followed by a multi-head self-attention module designed to capture the underlying relationships between individual CpG sites and the overall enhancer state. The output of the self-attention module was then passed on to a two-layer neural network, which served as the final classifier. The specific hyperparameters used in the model are shown (top right).

a

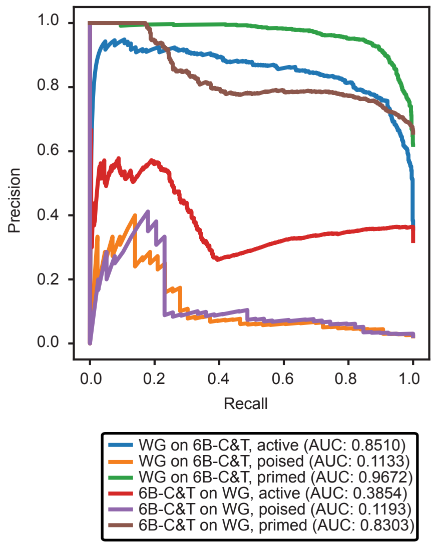

b

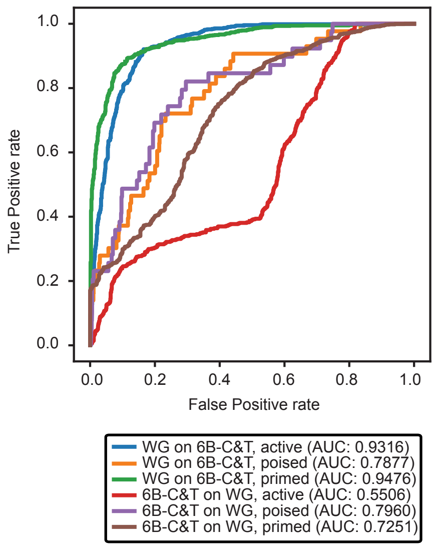

Supplementary Figure 18. Cross-dataset validation of 6B-C&T vs. WG 6B-seq machine learning models for different enhancer types. (a) Individual (single class) precision-recall curves for different training vs. test dataset combinations (e.g., whole genome on 6B-C&T: trained on whole genome data and tested on 6B-C&T data). Tests performed on chromosome 1 data (not included in training) using the trained model for classification into three enhancer types. **(b)** Individual (single class) Receiver Operating Characteristic curves for the machine learning framework described in **a**. Colour legend and AUC values are shown in the tables for each plot.

Supplementary references

1. Fullgrabe, J. et al. Simultaneous sequencing of genetic and epigenetic bases in DNA. *Nat Biotechnol* **41**, 1457-1464 (2023).
2. Li, R., Grimm, S.A. & Wade, P.A. CUT&Tag-BS for simultaneous profiling of histone modification and DNA methylation with high efficiency and low cost. *Cell Rep Methods* **1**, 100118 (2021).
3. Cruz-Molina, S. et al. PRC2 Facilitates the Regulatory Topology Required for Poised Enhancer Function during Pluripotent Stem Cell Differentiation. *Cell Stem Cell* **20**, 689-705 e689 (2017).
