# Supplementary material for: Sequencing DNA methylation and hydroxymethylation at co-occurring chromatin features
Source: Nat Commun. 2026 Feb 10;17:2591. doi: 10.1038/s41467-026-69429-6 (PMC13002996; doi:10.1038/s41467-026-69429-6)
Supplement: Supplementary file 2 — Description of Additional Supplementary Files [file 41467_2026_69429_MOESM2_ESM.pdf]

Description of Additional Supplementary Files

File name: Supplementary Data 1

Description: Supplementary Data Tables

Table legends:

Table 1 – Descriptive statistics for 5mCpG/5hmCpG analysis in Supplementary Figure 14
Table 2 – DNA oligonucleotides used in this study
Tables 3 – 6-base resolution stats for 6B-CUT&Tag datasets generated in this study - Summary results with scar filter
Table 4 – 6-base resolution stats for 6B-CUT&Tag datasets generated in this study - Summary results without scar filter
Table 5 – 6-base resolution stats for whole-genome 6-base-seq datasets generated in this study - Summary results (scar filter only)
Table 6 – 6-base resolution stats for IgG control 6B-CUT&Tag - Summary results with scar filter
Table 7 – 6-base resolution stats for IgG control 6B-CUT&Tag - Summary results without scar filter
Table 8 – Summary of findings and conclusions from Supplementary Figures 4, 9, 10, 14, 15, 16 and 18
